# Endoscopic Retrograde Cholangiopancreatography–Induced Splenic Injury in a Patient With Sleeve Gastrectomy

**DOI:** 10.1177/2324709618779417

**Published:** 2018-06-17

**Authors:** Laith Al Momani, Shoura Karar, Lindsey C. Shipley, Allison Locke, James Swenson

**Affiliations:** 1 East Tennessee State University, Johnson City, TN, USA; 2 University Hospital of Southampton, Southampton, UK

**Keywords:** endoscopic retrograde cholangiopancreatography, splenic injury, complications, sleeve gastrectomy

## Abstract

Endoscopic retrograde cholangiopancreatography (ERCP) is an invasive procedure with significant complications. Splenic hematoma is an extremely rare but known complication following ERCP that has been increasingly reported in the past several years. We report the case of a 44-year-old patient with a history of sleeve gastrectomy who underwent an ERCP that was complicated by both acute pancreatitis and splenic hematoma. She was managed conservatively under close monitoring in the intensive care unit. Clinicians should be aware of this potentially life-threatening complication to make a prompt diagnosis and begin early appropriate management.

## Introduction

Endoscopic retrograde cholangiopancreatography (ERCP) is currently being used more frequently for the diagnosis and treatment of complex pancreatico-biliary conditions. The procedure carries a rate of complications that varies between 5% and 10%.^1-3^ Complications include acute pancreatitis, which is the most common complication, hemorrhage, and duodenal or esophageal perforation. The procedure carries an overall mortality of 0.5% to 1%.^4^ Splenic injury, while a relatively rare complication of ERCP with only about 20 cases reported in the last 27 years, is potentially lethal. In this article, we present the case of a 44-year-old patient with a history of sleeve gastrectomy who underwent an ERCP that was complicated by both acute pancreatitis and splenic hematoma.

## Case Presentation

**Figure 1.**
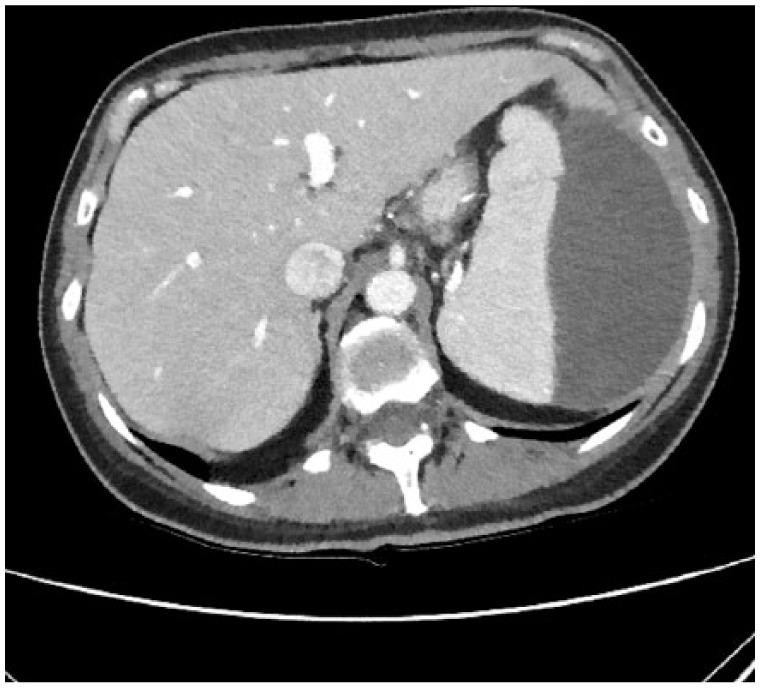
Computed tomography scan of the abdomen illustrating a large splenic heterogeneous subcapsular hematoma and peripancreatic stranding, transverse plane.

**Figure 2.**
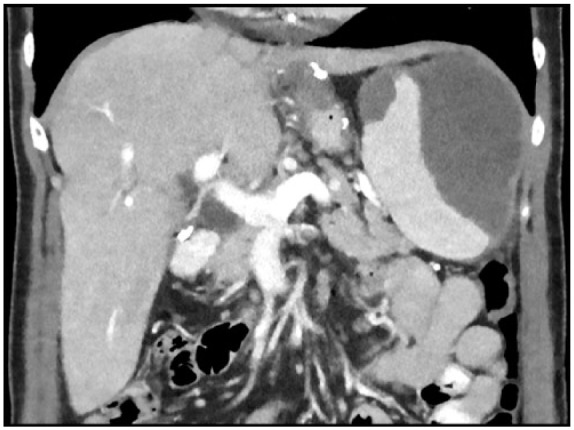
Computed tomography scan of the abdomen illustrating a large splenic heterogeneous subcapsular hematoma and peripancreatic stranding, coronal plane.

A 44-year-old woman presented to the emergency department with complaint of intractable nausea and vomiting associated with severe epigastric pain of 2 days duration. She denied any changes in her bowel habits. Review of systems was otherwise negative. She is status post sleeve gastrectomy 2 years prior and had a history of a remote laparoscopic cholecystectomy. Physical examination was only notable for mild epigastric tenderness. Laboratory tests showed aspartate aminotransferase 46 IU/L, alanine aminotransferase 65 IU/L, alkaline phosphatase 75 IU/L, and otherwise normal including lipase. A right upper quadrant ultrasound showed dilation of the common bile duct, which was again demonstrated on magnetic resonance cholangiopancreatography as well as a 7-mm common bile duct stone. The patient subsequently underwent ERCP with sphincterotomy. Cholangiogram at the time demonstrated a dilated common bile duct with no obvious cause. A few hours postoperatively, the patient started experiencing severe epigastric and left upper quadrant abdominal pain radiating to the back. She was found to be hypotensive and immediate fluid resuscitation was initiated. Repeat laboratory testing at the time showed a lipase of 1300 IU/L as well as a decline in hemoglobin from baseline of 12.0 g/dL to 7.0 g/dL. A CT (computed tomography) scan of the abdomen illustrated a large splenic heterogeneous subcapsular hematoma and peripancreatic stranding (Figures 1 and 2). The patient was transferred to the intensive care unit and managed conservatively with fluids and blood transfusions. The hematoma regressed, and her hemoglobin remained stable. With clinical improvement, she was discharged home.

## Discussion

Numerous cases of splenic injury after colonoscopy are described in the literature, but such injuries resulting from ERCP are quite rare. The first case reported of splenic injury following ERCP was by Trondsen et al^5^ in 1989, where a female patient presented with hemoperitoneum several hours after her ERCP due to a decapsulated spleen necessitating splenectomy. Similar cases have been reported since then.

Other rare complications of ERCP reported include liver laceration and disruption of the transverse mesocolon with resultant colonic ischemia.^6,7^ These rare complications, however, are not typically considered in the immediate postprocedure time frame.

Signs and symptoms that a clinician should be aware of in case of a possible splenic injury are not specific and considerably overlap with more common complications of ERCP, such as perforation, post–sphincterotomy bleeding, and pancreatitis. The typical presentation often includes left upper quadrant and epigastric abdominal pain with or without signs of peritonitis, hemodynamic instability, and/or markedly reduced hemoglobin with no hematemesis or melena.^8^ Delayed diagnosis is occasionally a characteristic feature as the appearance of symptoms following splenic injury may vary from a few hours to a few days. This was also attributed to the lack of awareness of this rare complication.^9^

A high index of suspicion is required to promptly diagnose this type of injury following ERCP. Although abdominal ultrasound scan has been used, CT scan is still considered the test of choice to investigate any suspected severe complication and often the exact diagnosis can only be established intraoperatively. Pathologic features commonly found on CT scan include free fluid (hemoperitoneum), subcapsular or perisplenic hematoma, and splenic laceration.^3,8,10^ Several findings of post-ERCP splenic injury have been described, including laceration, subcapsular hematoma, short gastric vessel avulsion, and direct tearing of the splenic capsule.^7,11,12^

The exact mechanism of splenic injury remains speculative. It is hypothesized that splenic injury occurs when the endoscope “bows” in the “long” position with torsion on the greater curvature of the stomach, while attempting to pass the scope through the duodenum, causing splenic capsular tears or vascular avulsion of the short gastric vessels.^1,13-15^ Calcification or fibrosis from chronic pancreatitis or adhesions from previous abdominal surgeries can make this portion of the procedure more difficult and hence require more traction. It has been suggested that a more complicated and prolonged procedure might increase the risk of a splenic injury.^13^ Furthermore, direct splenic trauma by the endoscope is also a possibility often resulting in injury to the medial surface of the spleen.^16^ Putting all of the above into prospective, it seems more often than not, this complication has been mostly reported in uncomplicated, uneventful procedures.^6^

To the best of our knowledge, of the minimal number of post-ERCP splenic injury case reports this is the first to be reported on a patient with a gastric sleeve. Correlating with the above possible explanations from the literature, we hypothesize that these patients are at an increased risk for a splenic injury due to traction. This might be due to the fibrotic changes complicating the laparoscopic procedure or resulting from exposing the greater curvature and separately coagulating the short gastric vessels at the time of the sleeve gastrectomy.^17^ We suggest that these patients might potentially be at an increased risk for this rare complication and exceptional care should be taken in the perioperative period.

Patients with potential post-ERCP splenic injury are managed based on their clinical state and hemodynamic stability. Most patients recover fully after receiving either splenectomy or conservative management. Despite different mechanisms of injury, the management remains similar as per any blunt splenic trauma, and hence, trauma guidelines should be followed. Early diagnosis may increase the likelihood of preserving the spleen.^8,18^

## Conclusion

In conclusion, splenic injury is a rare yet clinically significant complication of ERCP. We present a case of splenic lacerations that occurred in a patient with gastric sleeve. Increased awareness of such potentially life-threatening complication following ERCP, especially in patients who are predisposed to this complication like this particular patient, should decrease the incidence of adverse events and allow for early intervention when needed.
